# Sociology and Criminology

**Published:** 1896-01

**Authors:** 


### Sociology and Criminology.

The following are the opening remarks of Moritz Ellinger, Esq., Secretary of the International Medico-Legal Congress, Department of Sociology and Criminology, September 5, 1895: "Sociology and Criminology are sciences which have been placed in the front rank in this century of ours as the most comprehensive sciences touching us; affecting as they do the most vital interests of government and the progress of the social structure. Man in former years was only studied in his relation to the nearest surroundings, the family and the community in which he had his domicile, and the privileges which were accorded to him according to his rank, his station and occupation. It was due to the scientists like Herbert Spencer, Comte, Cæsare Lombroso, that the development was made the subject of study and the principles determining its progress defined with scientific accuracy, and which must be adhered to in order to conform with the unalterable laws which reign in the social as well as in the physical world. As I will read a paper on this subject I will not detain the Congress with any further remarks on this subject, but introduce Mr. Crothers, who is to make a few brief remarks."—*From advance sheets Medico-Legal Journal and Bulletin Medico-Legal Congress.*

